# Strain-specific pathogenicity and subversion of phenoloxidase activity in the mosquito *Aedes aegypti* by members of the fungal entomopathogenic genus *Isaria*

**DOI:** 10.1038/s41598-018-28210-6

**Published:** 2018-07-02

**Authors:** José L. Ramirez, Ephantus J. Muturi, Christopher Dunlap, Alejandro P. Rooney

**Affiliations:** Crop Bioprotection Research Unit, National Center for Agricultural Utilization Research, Agricultural Research Service, United States Department of Agriculture, Peoria, Illinois USA

## Abstract

Development of alternative vector control strategies are becoming more pressing given the rapid evolution of insecticide resistance and the rise of vector borne pathogens affecting public health such as dengue, chikungunya and Zika. Fungal-based biopesticides are promising alternatives to synthetic insecticides because they are ecofriendly and are highly effective at infecting insects through contact. This study evaluated the susceptibility of the yellow fever mosquito *Ae*. *aegypti* to a range of entomopathogenic fungal strains from the genus *Isaria*. We observed a diverse variation in the virulence of the *Isaria* strains tested, with two strains showing high pathogenicity towards adult mosquitoes. Mosquito susceptibility to fungal infection was further corroborated through the molecular quantification of fungal loads and the transcript evaluation of a fungal-specific pathogen recognition molecule in the mosquito body. Moreover, quantitative analysis of transcript abundance coupled with enzymatic assays revealed strain-specific subversion of the melanization cascade, an important immune response component. Our study contributes critical insights for a better understanding of fungal-mosquito interactions.

## Introduction

Fungal entomopathogens have attracted significant research interest as promising alternatives to chemical-based strategies for controlling mosquitoes and other vectors of human pathogens^1,2^. These efforts have become increasingly necessary due to the rising problem of insecticide-resistance in all major mosquito vectors^3–5^, and the rise of new and re-emergent vector borne pathogens affecting public health^6–9^.

Invasion of the insect body by fungal entomopathogens occur through specific biological adaptations that include the development of infection pegs and production of cuticle degrading enzymes that allow them to penetrate the arthropod chitinous exoskeleton^10^. Successful germination and infection depend on several factors that include host susceptibility, host life stage, and environmental conditions such as temperature and humidity^10^. Once inside the mosquito hemocoel, the invading fungus proliferate as hyphal bodies or blastospores, disseminating throughout the body of the mosquito until the host succumbs to the infection^10,11^. Host mortality is thought to occur from both mycotoxin production and the over proliferation of fungal bodies inside the insect host^10,12,13^.

As the fungal entomopathogen penetrates the mosquito body and makes its way towards the hemocoel, it faces potent cellular and humoral immune defenses mounted by the arthropod host as it attempts to overcome the infection^10,14^. Recognition of fungal components by pathogen recognition receptors, soon after infection, initiates the anti-fungal response through the induction of several immune pathways and production of antipathogenic effectors to counteract the infection. One such fungal-specific recognition molecule is TEP22, a thioester-containing protein that has also been found to be a critical anti-fungal immune effector^15^. Following fungal recognition, a complex cellular and systemic immune response, modulated in part by the Toll and the JAK-STAT immune signaling pathways, are elicited to counteract the invading fungus^16,17^. In Toll pathway activation, recognition of fungal cell elements by pattern recognition receptors activates the extracellular serine protease cascade that results in the cleavage of the cytokine Spätzle, which then serves as a ligand for the Toll receptor. This triggers the signaling cascade that leads to the phosphorylation and degradation of the negative regulator Cactus, freeing the NF-kB transcription factor Rel1 and allowing its nuclear translocation and the transcription of antimicrobial effectors^16,18^. The JAK-STAT pathway is known for its role in antiviral and antibacterial immunity but has also been recognized for its implication in the antifungal response^19–21^. Its elicitation, in response to injury or microbial infection, leads to signal transduction that culminates in the phosphorylation and nuclear translocation of the transcription factor STAT, and the induction of effector genes^22^. Two other mosquito innate immune signaling pathways (Imd and JNK pathways) are recognized for their role in the antibacterial and antiplasmodial response^23^ but their implication in the anti-fungal response is less known.

The melanization cascade is another important anti-fungal immune mechanism in mosquitoes, containing or limiting the spread of the fungal infection^24^. Recognition of fungal components by the mosquito’s pathogen pattern recognition receptors initiate this cascade by activating a series of serine proteases that in turn cleave and convert prophenoloxidase (PO) into active phenoloxidase (PO)^14,24^. Thus, prophenoloxidase enzymes (PPO) are key components catalyzing this cascade^25^, leading to melanin deposition, sequestration and encapsulation of the invading microbe^26^. In fact, several of these genes have been shown to be elicited following infection with the entomopathogenic fungi *Beauveria bassiana*^27,28^ highlighting their role in limiting fungal infections.

The genus *Isaria* is a widespread group of entomopathogenic fungi with diverse number of species^29^. Most of the *Isaria* strains have been found infecting larvae, pupae and adults of a wide range of insect orders, and a few of its members are currently being tested for their efficacy against several arthropod pests^30–32^. Although a few *Isaria* strains have been tested against insects, fewer studies have examined the susceptibility of mosquitoes to this entomopathogenic genus^33–35^.

This study focused on the genus *Isaria*, evaluating the pathogenicity of several strains against one of the most important arthropod vectors, the yellow fever mosquito *Ae*. *aegypti*. Our studies revealed contrasting effects of infection on longevity and fungal recognition by the mosquito body. Moreover, our results indicate a widespread absence of pro-phenoloxidase gene elicitation/transcription and strain-specific subversion of PO activity, major components of the melanization cascade. Our study contributes important new insights for a better understanding of fungal-mosquito interactions.

## Results

### Mosquito dose-response to diverse *Isaria spp*

**Figure 1:**
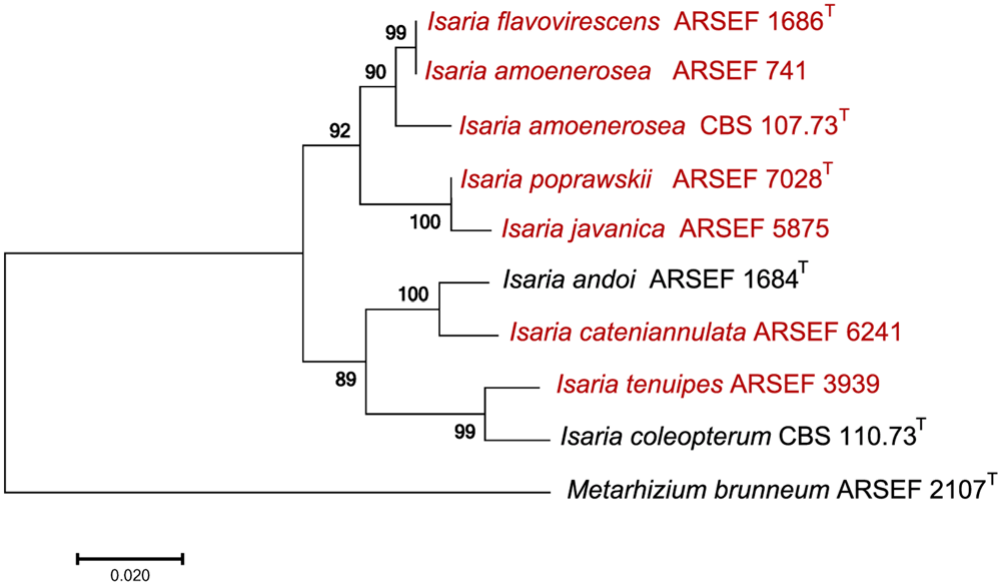
Phylogenetic relationships of *Isaria* strains used in this study in comparison to other reference strains. The phylogenetic analysis was based on 2 loci (*BTUB* and *TEF*) and conducted using the maximum likelihood method based on the Tamura-Nei model. Numbers along branches represent percent bootstrap support values generated from 1500 pseudo-replications. The scale bar at the bottom left represents 0.020 substitutions per nucleotide site.

In order to evaluate *Isaria* pathogenicity and dose-response in adult female *Ae*. *aegypti* mosquitoes, seven strains representing broad phylogenetic diversity within the genus were chosen for our study (Fig. 1). Strain ARSEF 5874 was accessioned as an *Isaria fumosorosea*, but was identified as an *Isaria javanica* in the current study. There have been several recent phylogenetic studies that show this error of *I*. *javanica* strains incorrectly reported as *I*. *fumosorosea* strains^32,36–38^.

**Figure 2:**
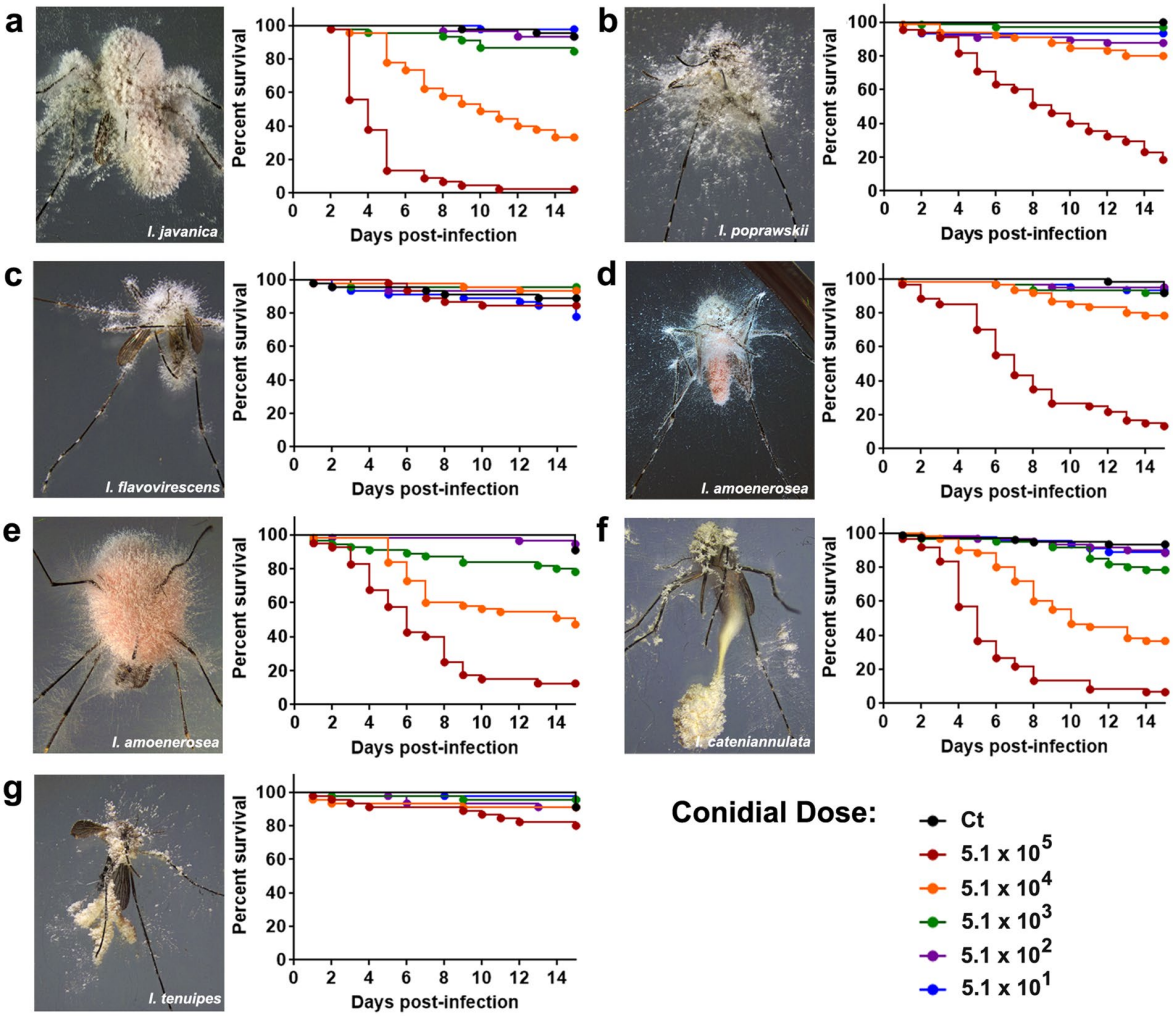
Survival curves and mycosed mosquito cadavers following infection by strains of the fungal entomopathogenic genus *Isaria*. (**a**) *I*. *javanica* ARSEF 5874, (**b**) *I*. *poprawskii* ARSEF 7028, (**c**) *I*. *flavovirescens* ARSEF 1686, (**d**) *I*. *amoenerosea* CBS 107.73, (**e**) *I*. *amoenerosea* ARSEF 741, (**f**) *I*. *cateniannulata* ARSEF 6241 and (**g**) *I*. *tenuipes* ARSEF 3939. Survival graphs represents 3 independent experiments and data was analyzed with Long-rank Test (GraphPad Prism 7).

The values for LC_50_ and LT_50_/LT_95_ were determined for each of the selected strains and varied among strains. *I*. *javanica* ARSEF 5874 and *I*. *cateniannulata* ARSEF 6241 showed the strongest dose-effect against adult *Ae*. *aegypti* mosquitoes (Fig. 2, Table 1). The LC_50_ and LT_50_ values for *I*. *tenuipes* ARSEF 3939 and *I*. *flavovirescens* ARSEF 1686 was not assessed due to mosquito survival above 50% on the last day of the experiment.

**Table 1:** Calculated LC_50_ (day 9 PI), LT_50_ and LT_95_ values on the highest fungal isolate dose (conidia/mosquito) used against adult *Aedes aegypti*.

Fungal Isolate	LC_50_ (95% CI)	LT_50_ (95% CI)	LT_95_ (95% CI)
*I*. *tenuipes* (ARSEF 3939)	ND	ND	ND
*I*. *amoenerosea* (CBS 107.73)	2.2 × 10^6^ (5.8 × 10^5^–2.1 × 10^7^)	10.28 (9.77–10.83)	ND
*I*. *javanica* (ARSEF 5875)	4.2 × 10^4^ (2.7 × 10^4^–7.0 × 10^4^)	3.93 (3.44–4.38)	9.06 (8.42–9.86)
*I*. *cateniannulata* (ARSEF 6241)	5.1 × 10^4^ (3.1 × 10^4^–9.1 × 10^4^)	5.14 (4.71–5.54)	11.18 (10.55–11.95)
*I*. *amoenerosea* (ARSEF 741)	9.9 × 10^4^ (6.2 × 10^4^–1.7 × 10^5^)	7.48 (7.03–7.92)	ND
*I*. *flavovirescens* (ARSEF 1686)	ND	ND	ND
*I*. *poprawskii* (ARSEF 7028)	7.3 × 10^5^ (3.4 × 10^5^–2.9 × 10^6^)	10.18 (9.81–10.58)	ND

ND (not determined) indicates that values could not be calculated because mortality was lower than 50% or 95% at the end of the experiment.

### Mosquito Survival Post-fungal infection

Mosquito survival post fungal infection differed with each fungal strain and with each fungal dose used in the infection bioassays (Fig. 2). Survival curves differed significantly from the control group for mosquitoes infected with *Isaria javanica* ARSEF 5874 (log-rank Mantel–Cox test, dose 5.1 × 10^5^, *χ*^2^: 96.09, *P* < 0.0001 and dose 5.1 × 10^4^, *χ*^2^: 38.15, *P* < 0.0001); *I*. *poprawskii* ARSEF 7028 (log-rank Mantel–Cox test, dose 5.1 × 10^5^, *χ*^2^: 96.68, *P* < 0.0001 and dose 5.1 × 10^4^, *χ*^2^: 14.36, *P* = 0.0002); *I*. *amoenerosea* CBS 107.73 (log-rank Mantel–Cox test, dose 5.1 × 10^5^, *χ*^2^: 91.43, *P* < 0.0001 and dose 5.1 × 10^4^, *χ*^2^: 4.753, *P* = 0.0292); *I*. *amoenerosea* ARSEF 741 (log-rank Mantel–Cox test, dose 5.1 × 10^5^, *χ*^2^: 82.3, *P* < 0.0001 and dose 5.1 × 10^4^, *χ*^2^: 27.9, *P* < 0.0001); and *I*. *cateniannulata* ARSEF 6241 (log-rank Mantel–Cox test, dose 5.1 × 10^5^, *χ*^2^: 121.1, *P* < 0.0001, dose 5.1 × 10^4^, *χ*^2^: 50.69, *P* < 0.0001 and dose 5.1 × 10^3^, *χ*^2^: 6.166, *P* = 0.0130). Two fungi, *I*. *tenuipes* ARSEF 3939 (log-rank Mantel–Cox test, dose 5.1 × 10^5^, *χ*^2^: 3.667, *P* = 0.1024) and *I*. *flavovirescens* ARSEF 1686 (log-rank Mantel–Cox test, 5.1 × 10^5^, *χ*^2^: 0.3678, P = 0.5442) did not significantly affect mosquito survival with the range of doses tested (Fig. 2).

We observed a marked variation in the proportion of mosquito cadavers that successfully sporulated among all treatments. The greatest numbers of sporulated mosquito cadavers were observed with the most virulent entomopathogenic fungi; *I*. *javanica* ARSEF 5874 (87%), *I*. *amoenerosea* CBS 107.73 (84%) and *I*. *cateniannulata* ARSEF 6241 (83%). The sporulation rate of mosquito cadavers was lower in mosquitoes infected with *I*. *tenuipes* ARSEF 3939 (59%), *I*. *amoenerosea* ARSEF 741 (62%), *I*. *flavovirescens* ARSEF 1686 (52%), and *I*. *poprawskii* ARSEF 7028 (75%). Furthermore, although higher doses led to higher mosquito mortality it did not translate into a higher success for mycelial growth/sporulation since cadavers from lower doses attained higher sporulation percentages. This was especially true for the most pathogenic strains (Table S.1). Although, a few control mosquitoes perished during the span of the experiments, none of them presented evidence of fungal infection/sporulation.

### Fungal loads post-infection and immune fungal recognition by the mosquito

**Figure 3:**
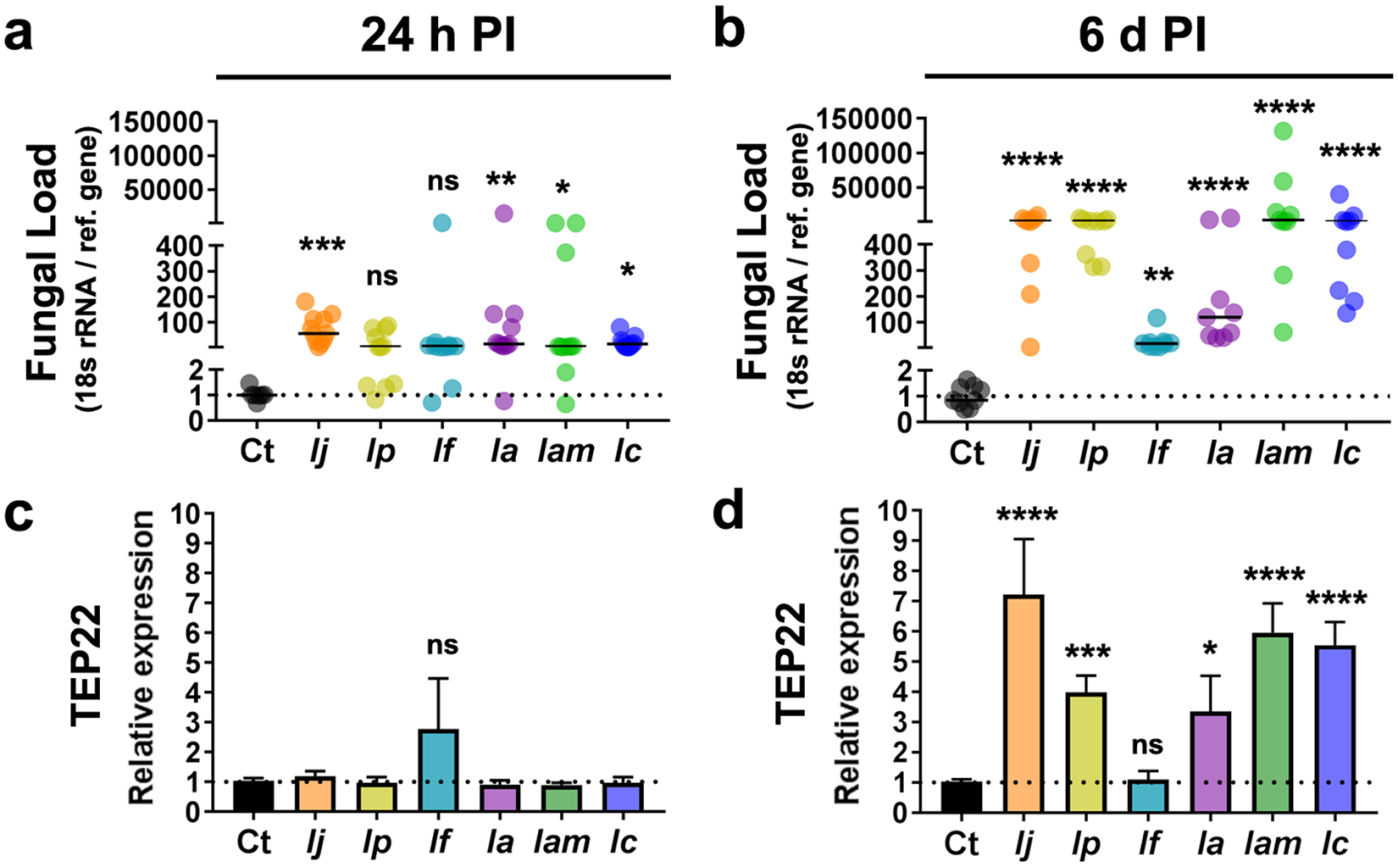
The mosquito supports fungal proliferation and is able to recognize infection by strains of the fungal entomopathogenic genus *Isaria*. Fungal loads via relative quantification of fungal 18 s rRNA in the whole body of mosquitoes at (**a**) 24 h PI and (**b**) 6d PI. Each dot represents the fungal load value from a pool of 5 mosquitoes and the horizontal black bar indicates the median fungal load. Relative expression of the fungal recognition and fungal effector gene *TEP22* in the whole body of mosquitoes at (**c**) 24 h PI and (**d**) 6d PI. Data represents the fold change in expression from 3 independent experiments. The statistical significance of fold change values was determined on log_2_ transformed values via one-way ANOVA with Dunnett’s post-test; *P < 0.05, **P < 0.01, ***P < 0.001, ****P < 0.0001, ns = not-significant. Error bars indicate the SEM of three independent experiments. *Ij* = *I*. *javanica* ARSEF 5874, *Ip* = *I*. *poprawskii* ARSEF 7028, *If* = *I*. *flavovirescens* ARSEF 1686, *Ia* = *I*. *amoenerosea* CBS 107.73, *Iam* = *I*. *amoenerosea* ARSEF 741, and *Ic* = *I*. *cateniannulata* ARSEF 624.

To confirm fungal amplification post-infection, we molecularly evaluated the fungal load in mosquitoes challenged with the most virulent fungi at 24 h and 6d post-infection (PI). As a control we also included one of the fungal strains that did not have a significant effect on mosquito mortality. Fungal load was conducted by analyzing the transcript abundance of the fungal 18 s rRNA gene using primers that were designed to amplify a highly conserved region of the gene among fungi^39^. Our analyses confirmed significantly higher amounts of fungal genomes in mosquitoes challenged with the fungal entomopathogens in comparison with low levels of natural fungal background found in the control group especially at 6d PI (Fig. 3a,b). The fungal genomes found in the highest amounts, in relation to the control group at 6d PI, were *I*. *javanica* ARSEF 5874 (ANOVA, Dunnett’s test, *P* = 0.0001), *I*. *poprawskii* ARSEF 7028 (ANOVA, Dunnett’s test, *P* = 0.0001), *I*. *amoenerosea* ARSEF 741 (ANOVA, Dunnett’s test, *P* = 0.0001), *I*. *amoenerosea* CBS 107.73 (ANOVA, Dunnett’s test, *P* = 0.0001) and *I*. *cateniannulata* ARSEF 6241 (ANOVA, Dunnett’s test, *P* = 0.0001). Lower levels, albeit still significant, were found in mosquitoes infected with *I*. *flavovirescens* ARSEF 1686 (ANOVA, Dunnett’s test, *P* = 0.0035). Confirmation of fungal infection was conducted by assessing the transcript abundance of the mosquito gene *TEP22* in the whole body of *Ae*. *aegypti* mosquitoes at 24 h and 6d post-infection. *TEP22*, a thioester-containing protein, has been shown to specifically recognize fungal cells in the mosquito, where it also acts as an anti-fungal effector^15^. Its elicitation as a response to fungal invasion allows us to use it as a marker of successful fungal infection/colonization of the mosquito body. Although *TEP22* elicitation was not observed at 24 h PI, it was highly upregulated at 6d PI reflecting somewhat the fungal loads observed in the same cohort of mosquitoes at this time point of infection (Fig. 3c,d). The highest expression of *TEP22* was observed with *I*. *javanica* ARSEF 5874 (ANOVA, Dunnett’s test, *P* = 0.0001), *I*. *poprawskii* ARSEF 7028 (ANOVA, Dunnett’s test, *P* = 0.0005), *I*. *amoenerosea* ARSEF 741 (ANOVA, Dunnett’s test, *P* = 0.0001), and *I*. *cateniannulata* ARSEF 6241 (ANOVA, Dunnett’s test, *P* = 0.0001). A lower *TEP22* expression profile, albeit still significant, was seen in mosquitoes infected with *I*. *amoenerosea* CBS 107–73 (ANOVA, Dunnett’s test, *P* = 0.0255). In turn, mosquitoes infected with the least pathogenic fungal strain, *I*. *flavovirescens* ARSEF 1686, showed no significant increase in *TEP22* transcript abundance (ANOVA, Dunnett’s test, *P* = 0.9998); this despite showing significantly higher numbers of fungal genomes with respect to the control group (Fig. 3d).

**Figure 4:**
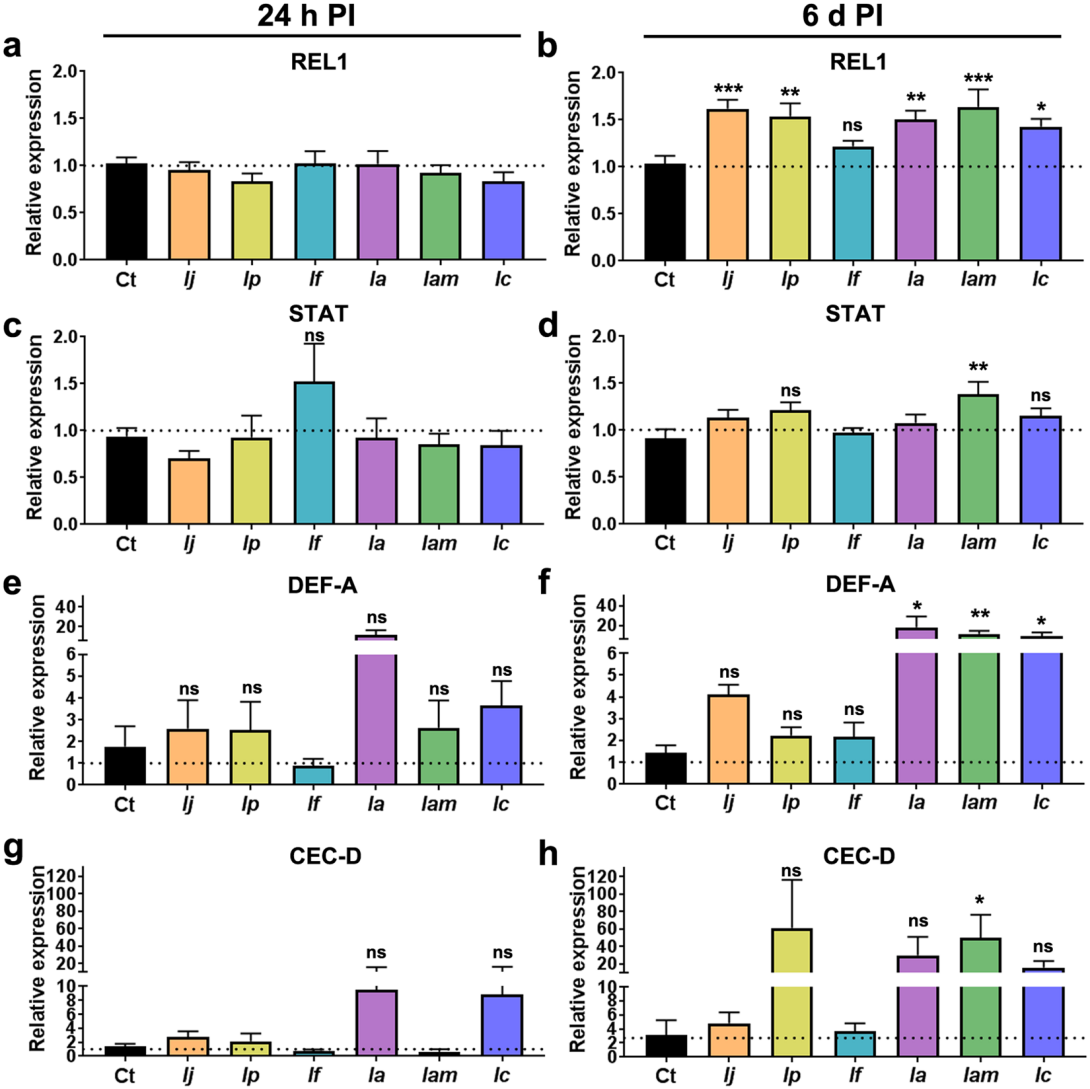
Elicitation of Toll and JAK-STAT pathways following fungal infection is time and fungal strain-specific. Panels represents the gene expression profiles of transcription factors *REL1* (Toll pathway) and *STAT* (JAK-STAT pathway), and the antimicrobial peptides defensin A (*DEF-A*) and cecropin D (*CEC-D*) in mosquito cohorts infected with *Ij* = *I*. *javanica* ARSEF 5874, *Ip* = *I*. *poprawskii* ARSEF 7028, *If* = *I*. *flavovirescens* ARSEF 1686, *Ia* = *I*. *amoenerosea* CBS 107.73, *Iam* = *I*. *amoenerosea* ARSEF 741, and *Ic* = *I*. *cateniannulata* ARSEF 624 at 24 h PI (**a**,**c**,**e** and **g**) and at 6d PI (**b**,**d**,**f** and **h**). Expression data (bar plots) represents the fold change in expression from three independent experiments. The error bars indicate the SEM and the statistical significance of fold change values was determined on log_2_ transformed values via one-way ANOVA with Dunnett’s post-test. *P < 0.05, **P < 0.01, ***P < 0.001, ns = not-significant.

Expression analyses of other important immune genes showed no significant modulation at the early stages of infection (24 h) but a significant upregulation at late stages of infection (6d PI, Fig. 4). Particularly notable were the elicitation of the Toll pathway transcription factor *REL1*, whose expression was highly elicited in mosquitoes infected with *I*. *javanica* ARSEF 5874 (ANOVA, Dunnett’s test, *P* = 0.0004), *I*. *poprawskii* ARSEF 7028 (ANOVA, Dunnett’s test, *P* = 0.0021), *I*. *amoenerosea* CBS 107-73 (ANOVA, Dunnett’s test, *P* = 0.0026), *I*. *amoenerosea* ARSEF 741 (ANOVA, Dunnett’s test, *P* = 0.0004), and *I*. *cateniannulata* ARSEF 6241 (ANOVA, Dunnett’s test, *P* = 0.01). No significant regulation in *REL1* expression was observed in mosquitoes infected with *I*. *flavovirescens* ARSEF 1686 (ANOVA, Dunnett’s test, *P* = 0.306) (Fig. 4a,b).

Our expression analysis of the JAK—STAT transcription factor STAT showed less infection-responsive regulation, with only mosquitoes infected with *I*. *amoenerosea* ARSEF 741 presenting significant STAT upregulation (ANOVA, Dunnett’s test, *P* = 0.0096) (Fig. 4c,d). In addition, our analysis of two antimicrobial peptides, defensin (*DEF-A*) and cecropin (*CEC-D*), previously shown to be elicited during fungal infection showed no significant regulation at 24 h PI but presented varying levels of expression at 6d (Fig. 4e–h). Particularly notable at 6d PI was the upregulation of defensin A (*DEF-A*) in mosquitoes infected with *I*. *amoenerosea* CBS 107-73 (ANOVA, Dunnett’s test, *P* = 0.042), *I*. *amoenerosea* ARSEF 741 (ANOVA, Dunnett’s test, *P* = 0.0045), and *I*. *cateniannulata* ARSEF 6241 (ANOVA, Dunnett’s test, *P* = 0.0223) (Fig. 4e,f). Cecropin (*CEC-D*) expression was more irregular at this late stage of infection with only *I*. *amoenerosea* ARSEF 741 presenting a significant upregulation (ANOVA, Dunnett’s test, *P* = 0.0428) (Fig. 4h).

### Entomopathogenic fungal infection leads to a decrease in phenoloxidase activity and downregulation of PPO gene expression

**Figure 5:**
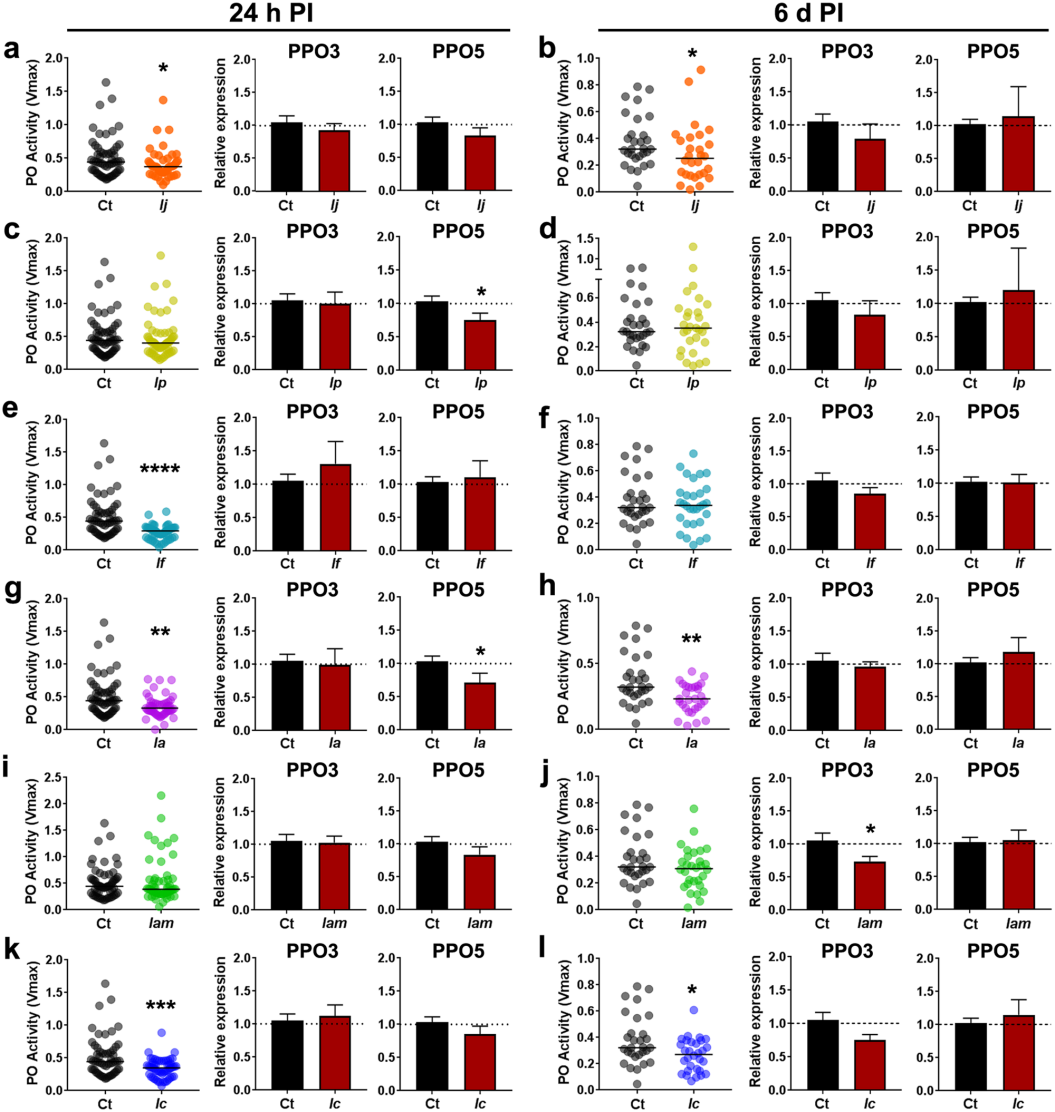
Fungal infection by members of the *Isaria* genus fail to elicit PPO gene regulation and leads to strain-specific reduction of phenoloxidase (PO) activity. Panels represents the phenoloxidase activity and gene expression profiles of PPO3 and PPO5 in mosquito cohorts infected with *Ij* = *I*. *javanica* ARSEF 5874, *Ip* = *I*. *poprawskii* ARSEF 7028, *If* = *I*. *flavovirescens* ARSEF 1686, *Ia* = *I*. *amoenerosea* CBS 107.73, *Iam* = *I*. *amoenerosea* ARSEF 741, and *Ic* = *I*. *cateniannulata* ARSEF 624. Phenoloxidase activity (Vmax) was evaluated from whole-body macerates of mosquitoes at 24 h and 6d PI. Each dot represents a pool of two mosquitoes and the horizontal bar indicates the median level of PO activity. Data represents samples from three independent experiments. Data was analyzed via Mann-Whitney test *P < 0.05, **P < 0.01, ***P < 0.001. Expression data (bar plots) represents the fold change in expression from three independent experiments. The error bars indicate the SEM and the statistical significance of fold change values was determined on log_2_ transformed values via student unpaired *t*-test. *P < 0.05, **P < 0.01, ***P < 0.001.

Given that the melanization cascade has been implicated in the mosquito antifungal defense system^24^, we selected the most pathogenic fungi and one of the least pathogenic strains (from our screen above) to further assess the effects of fungal infection on the elicitation of this immune response cascade. The first step was to look at the whole-body expression of *PPO3* and *PPO5*, important members of the phenoloxidase cascade at 24 h and 6d post-infection. Our gene expression analysis did not show significant regulation with any of the fungal strain challenges for PPO3 expression at 24 h PI and was only significantly downregulated at 6d PI in *I*. *amoenerosea* ARSEF 741-challenged mosquitoes (Fig. 5a,c,e,g,i and k). Infections with four other fungi led to a slight PPO3 downregulation at 6d PI but they were not significant from the control (Fig. 5b,d,f,h,j and l). This was the case for mosquitoes infected with *I*. *javanica* ARSEF 5874, *I poprawskii* ARSEF 7028, *I*. *amoenerosea* ARSEF 741 and *I*. *cateniannulata* ARSEF 6241, Similar results were observed for PPO5 expression, with significant PPO5 downregulation in mosquitoes infected with *I poprawskii* ARSEF 7028 and *I*. *amoenerosea* CBS 107.73 at 24 h PI but with transcript regulation indistinguishable from the control group at 6d PI (Fig. 5). To corroborate this finding, we decided to measure the phenoloxidase (PO) enzymatic activity in mosquito whole bodies at 24 h and 6d post-infection. Interestingly, we observed a drastic decrease in PO activity at 24 h PI in mosquitoes challenged with *I*. *javanica* ARSEF 5874 (Mann Whitney test, *P* = 0.0332), *I*. *flavovirescens* ARSEF 1686 (Mann Whitney test, *P* = 0.0001), *I*. *amoenerosea* CBS 107.73 (Mann Whitney test, *P* = 0.0012) and with *I*. *cateniannulata* ARSEF 6241 (Mann Whitney test, *P* = 0.0008). This decline in PO activity was maintained up to 6d post-infection in mosquitoes challenged with *I*. *javanica* ARSEF 5874 (Mann Whitney test, *P* = 0.0450), *I*. *amoenerosea* CBS 107.73 (Mann Whitney test, *P* = 0.0039), and with *I*. *cateniannulata* ARSEF 6241 (Mann Whitney test, *P* = 0.0233). In comparison, mosquitoes infected with *I*. *flavovirescens* ARSEF 1686 (Mann Whitney test, *P* = 0.7778) no longer showed a decline in PO activity and no significant changes were observed in the remaining mosquito groups infected with *I*. *amoenerosea* ARSEF 741 (Mann Whitney test, *P* = 0.2528), or with *I*. *poprawskii* ARSEF 7028 (Mann Whitney test, *P* = 0.9477) (Fig. 5).

## Discussion

Entomopathogenic fungi differ in their ability to infect and colonize their arthropod hosts. Our comparative study testing the pathogenicity of several *Isaria* strains against the adult *Ae*. *aegypti* mosquito, highlighted these differences. Five out of the seven fungal strains tested were observed to significantly affect mosquito survival. In this case, mortality of infected mosquitoes started to occur at 3d PI and gradually increased until the end of the trial for *I*. *javanica* ARSEF 5874, *I*. *amoenerosea* CBS 107.73, *I*. *cateniannulata* ARSEF 6241, *I*. *amoenerosea* ARSEF 741, and *I*. *poprawskii* ARSEF 7028 (15d PI). However, among all *Isaria* strains tested, *I*. *javanica* ARSEF 5874 and *I*. *cateniannulata* ARSEF 6241 were the most rapid in effecting a host response. These strains significantly reduced mosquito longevity with an LT_50_ of 3.9 and 5.1 days PI respectively, following infection with 5.1 × 10^5^ conidia/mosquito. In addition to the reduction in mosquito longevity, fungal entomopathogens have been shown to exert detrimental effects on other mosquito biology parameters such as fecundity and feeding success^40–42^. Hence, although the remaining fungal strains had moderate to marginal pathogenicity, it is quite possible that they would still have an impact on other mosquito physiological aspects that in turn affects their vectorial capacity.

The sporulation of fungi emerging from mosquito cadavers confirmed the successful infection and completion of the fungal life cycle in mosquitoes. Interestingly, the highest sporulation rates did not necessarily occur in the groups infected with the highest doses. This was especially true for the most pathogenic strains, *I*. *javanica* ARSEF 5874 and *I*. *cateniannulata* ARSEF 6241, in which 100% sporulation was observed in mosquito cadavers that resulted from a lower infection dose (Table S1). Although this study did not focus on fungal growth dynamics, this observation might be a reflection of further fungal adaptations to the insect host.

The fungal load analysis for each of our treatment groups corroborated our mosquito survival data, as the treatment groups with the highest mortality also portrayed high relative amounts of fungal genomes at 6d PI. In a deviation from what has been observed in *B*. *bassiana* infection^28^, the *Isaria* strains tested in this study did not elicit *TEP22* expression at 24 h PI but showed high significant induction at 6d PI. This likely indicates variation in host responses to the fungal infection or strain-specific dynamics of infection. The significant high expression of *TEP22* in five of the treatment groups indicated successful fungal penetration and active replication of these fungi inside the mosquito body at 6d PI. Thus, our results are consistent with the fact that TEP22 is an important fungal sensor and anti-fungal effector, and that it serves as a marker of *Isaria* infection. Mosquitoes challenged with *I*. *flavovirescens* ARSEF 1686, a fungal strain with minimal pathogenicity to mosquitoes, showed the lowest amounts of fungal loads amongst all strains tested. The relatively low amounts of *I*. *flavovirescens* ARSEF 1686, despite being significantly higher than the background, could mean a slow fungal replication without detrimental effects on mosquito survival. However, expression of *TEP22* in this cohort of mosquitoes was not significantly different from that of the uninfected control, suggesting that this fungus was unable to completely penetrate the mosquito cuticle or that fungal infection was controlled early in the infection process.

Our analysis of the two canonical immune signaling pathways that have been implicated in the anti-fungal response, Toll and JAK-STAT pathway, showed temporal and strain-specific gene modulation. For instance, albeit the transcription factor *REL1* (Toll pathway) was not regulated at 24 h PI, it was significantly elicited at 6d PI for five of the *Isaria* strains. This *REL1* modulation coincided with those mosquitoes supporting high fungal load burden. In contrast, only mosquitoes infected with *I*. *amoenerosea* ARSEF-741 showed significant induction of the transcription factor *STAT* (JAK-STAT pathway), further indicating strain-specific fungal-mosquito interactions.

The divergent modulation of two antimicrobial peptides, defensin (*DEF-A*) and cecropin (*CEC-D*), also indicates temporal and *Isaria* strain-specific immune elicitation in the mosquito body. The lack of significant modulation of these two antimicrobial peptides in mosquitoes supporting high fungal loads at the later stages of infection might suggest immune suppression by some of these fungal strains but more studies are needed to conclusively define this possibility.

Another important finding of our study was the absence of elicitation or the downregulation of the melanization cascade. In fact, the melanization pathway has been recognized as an important immune response mechanism in arthropods and has been shown to play a critical role in the antifungal defense of mosquitoes^24,43,44^. Prior studies of *Ae*. *aegypti* infected with *Beauveria bassiana* have found increased expressions of PPO3 and PPO5 at 24 h post-infection^15^. Our expression analysis of these two PPO genes, which are critical components of the melanization cascade, displayed no major significant regulation in gene expression relative to the control group at 24 h or 6d post-infection. In addition, our studies also point to potential active suppression of this immune mechanism given that our assessment of PO enzymatic activity showed a decline in PO activity in mosquitoes challenged with the most pathogenic *Isaria* strains. The absence of significant PPO gene regulation but significant decrease in basal levels of PO activity relative to the control, might indicate that the mechanism of suppression/evasion are acting both upstream and downstream from PPO generation. Similar declines in PO enzymatic activity have been observed in other arthropods when infected with the fungal entomopathogen *B*. *bassiana*, such as in the case of the moth *Spodoptera litura*, the wax worm *Galleria mellonella* and in the dipteran host *Drosophila melanogaster*^44,45^. This active suppression of PO activity by *B*. *bassiana* appears to be in part due to the action of a secondary metabolite, (oosporein) produced soon after infection^46^. Whether the same mechanism used by *B*. *bassiana* is employed by the *Isaria* strains used in this study remains to be elucidated. The significant drop in PO enzymatic activity in *I*. *flavovirescens* ARSEF1686-infected mosquitoes at 24 h PI but absent at the later stages of infection might represent its inability to overcome other immune responses or to adjust to the mosquito environment. Hence, despite having the ability to suppress PO activity, other parameters of host susceptibility might have been prevented *I*. *flavovirescens* from establishing an infection.

Furthermore, the fact that this significant decline in PO enzymatic activity at the later stages of infection was not seen in all the *Isaria* strains that successfully infected the mosquito, indicates that they also differ in their effectiveness to actively suppress the melanization cascade. This could also suggest that maintenance of suppression of PO activity throughout the infection is also necessary for successful completion of the fungal life cycle. This could, in turn, explain the limited pathogenicity of some *Isaria* strains to *Ae*. *aegypti* in comparison to the most virulent strains. Although several factors have been accounted for the reduction in mosquito survival following fungal entomopathogenic infection^12,13,47^, the evasion/suppression of the melanization cascade by the *Isaria* would allow for a faster proliferation of blastospores thus contributing to the negative impact of fungal infection on mosquito survival. In fact, prior research with the mosquito *Anopheles gambiae* revealed that while the melanization response did not abort the growth of the fungus *B*. *bassiana* it retarded its proliferation significantly^48^.

In summary, this work demonstrates the relative susceptibility of *Ae*. *aegypti* to seven different *Isaria* fungal strains, with two strains presenting high levels of pathogenicity. Our expression analysis of TEP22 as a marker of fungal infection indicated successful *Isaria* infection, with substantial increase in fungal genomes in at least six of the strains. Finally, our study revealed an interesting dynamic of mosquito-fungal interaction with fungal infection leading to immune modulation of the Toll pathway at the late stages of infection, and occurring without the elicitation of the phenoloxidase pathway or with a significant drop in PO activity. This significant variation in PPO expression and PO activity reflects in part the fungal-specific interaction with this immune response mechanism. Additional studies are necessary to conclusively determine whether the *Isaria* strains tested are actively suppressing or eluding this mechanism of defense in the same manner as some *B*. *bassiana* strains.

## Materials and Methods

### Mosquito rearing

The *Aedes aegypti* Rockefeller strain was reared in standard insectary conditions at 28 °C, with a relative humidity of 70–80%, and a 12 h light/dark cycle. Adult mosquitoes were maintained on a 10% sucrose solution while larvae were reared on a mixture of rabbit food and tropical fish food. All experimental assays were conducted using mosquitoes that were three to five-days old.

### Fungal strains and infection assays

Seven strains of *Isaria* were used in this study: *I*. *javanica* ARSEF 5874, *I*. *poprawskii* ARSEF 7028, *I*. *flavovirescens* ARSEF 1686, *I*. *amoenerosea* CBS 107.73, *I*. *amoenerosea* ARSEF 741, *I*. *cateniannulata* ARSEF 6241 and *I*. *tenuipes* ARSEF 3939. Fungal isolates were grown on ¼ strength Sabouraud dextrose agar and yeast extract (SDAY) medium. Spore oil formulations were prepared with soy bean oil and conidia harvested from cultures maintained for 15 days at 26 °C. The conidial suspensions were briefly homogenized with an electronic pestle and filtered trough a cheese cloth to remove mycelia. Soy bean oil is known as an effective carrier, allowing attachment of the conidial formulation to the hydrophobic insect cuticle^49,50^. Conidial concentrations were determined using an improved Neubauer hemocytometer and adjusted to a desired concentration. Five different concentrations were used in this study and ranged from 1 × 10^5^ conidia/mL to 1 × 10^9^ conidia/mL. Infection assays were conducted by topically applying 50.6 nl of the conidial suspension to the ventral surface of the coxal region of cold-anesthetized mosquitoes using a Nanoject II micropipet. This corresponded to an estimated range of 50 to 506,000 conidia deposited per mosquito. The control group consisted of mosquitoes exposed to the same volume of soy bean oil absent of fungal conidia. At least three independent experiments were conducted for each assay using fresh conidial suspensions and new batches of mosquitoes for each experiment. Conidial viability was greater than 94% as assessed by plating on SDAY and examining at 24 h post-inoculation at 26 °C as previously described^51^. Following the infection assay, mosquitoes were transferred to an insect cage, maintained under standard insectary conditions and provided with 10% sucrose solution for the duration of the experiment. Survival was monitored daily, and all mosquito cadavers were removed from the cages and transferred to individual sterile petri dishes holding a moist filter paper to check for fungal growth. Mosquito survival data was analyzed using Kaplan-Meier survival analysis with median survival time differences between treatments compared using the Log-rank test (GraphPad 7.0). In addition, the LC_50_, LT_50_ and LT_95_ values were calculated by probit analysis using SAS 9.4 statistical package. Mosquito mortality in the control groups ranged from 0 to 20% and was corrected using Abbott’s formula^52^.

### Molecular Identification of Fungal Strains

To corroborate the true fungal identity of our strains we conducted a molecular identification by amplifying and sequencing partial segments of the Translation elongation factor (TEF) and β-tubulin (BTUB) genes. DNA was extracted from fungal hyphae via the CTAB method (Sigma) and polymerase chain reaction (PCR) amplification conducted using the AmpliTaq Gold 360 Master Mix (Applied Biosystems), ~100 ng template DNA, and 0.2 μM each of primers 983 f and 1567r for TEF^53^, or bt2a and bt2b for BTUB^54^. The PCR reactions included an initial denaturation step of 10 min at 95 °C followed by 35 cycles, each consisting of a 30 s denaturation step at 95 °C, a 30 s annealing step at 55 °C, and a 1 min extension step at 72 °C, and then followed by a final extension step for 7 min at 72 °C. Purification of amplicons were conducted using the Montage PCR Cleanup Filter Plates (Millipore) while sequencing was done using the ABI BigDye version 3.0 sequencing kit (Applied Biosystems, Foster City, CA). These products were purified with the BigDye XTerminator® Purification Kit (Applied Biosystems) at one-tenth the recommended volume and sequenced on an ABI3730 genetic analyzer (Applied Biosystems) according to the manufacturer’s suggested protocol. Sequences were assembled using Sequencer® 5.4 software (Gene Codes Corporation) and visually edited for accuracy. The CLCBio genomics workbench 10.0 software (Qiagen Inc.) was used to align the consensus sequences, while MEGA 7^55^ was used to conduct a phylogenetic analysis using the maximum likelihood method with a Tamura-Nei model and a nearest neighbor interchange heuristic search method. The concatenated alignments of 2 partial loci BTUB, β-tubulin (321 bp) and TEF, Translation elongation factor (930 bp) were used to determine the phylogenetic analysis. The level of bootstrap support was calculated from 1500 replicates (Fig. 1).

### Gene Expression

For gene expression assays, mosquitoes were inoculated as stated above with an equivalent of 5.1 × 10^4^ conidia per mosquito and three pools of five mosquitoes were collected at 24 h and 6d PI. Collection at 24 h PI was conducted to capture the initial interactions that occur at the very early stages of fungal infection, while collection at 6d PI was done to assess mosquito-fungal interactions at the late stages of infection, when mosquitoes are supporting high fungal loads. This late stage would give us insights to the immune status immediately preceding mosquito mortality. Sample RNA extraction was conducted via TRIzol (Invitrogen) according to the manufacturer’s instructions. RNA concentrations and quality were assessed via NanoDrop (Thermo Scientific), and cDNA synthesis was conducted on normalized amounts of RNA using the QuantiTect reverse transcription kit with DNA Wipeout (Qiagen). Quantitative real-time PCR was conducted in a 10 µl reaction using the PowerUp SYBR green Master mix qPCR kit (Qiagen) with gene specific primers (Table S2), and one microliter of the generated cDNA. Cycling conditions for the qPCR reaction consisted of a holding stage at 95.0 °C for 10 min and 40 cycles of 15 s at 95.0 °C and 1 minute at 60 °C. For each experiment, the expression profile was assessed in three pools per treatment group and each experimental assay was evaluated via three independent experiments, each conducted with new batches of mosquitoes and fresh conidial suspensions. Each sample was analyzed in duplicate and the expression level of target genes was normalized using a combination of two reference genes (the ribosomal protein Rps49/L32 gene, AAEL003396, and the ribosomal protein Rps17, AAEL004175)^56^ (Table S2). These two reference genes have been routinely used in expression profiles involving *Aedes*. *aegypti*^56,57^; including transcriptomic studies on mosquito-fungal interactions^13,47^. Fungal load was conducted by analyzing the transcript abundance of the 18s rRNA gene using fungal-specific primers (Table S2) designed to amplify a highly conserved region among fungi^39^. qPCR was conducted on an Applied Biosystems 5700 Fast Real-time PCR (Applied Biosystems) while gene expression profiles were evaluated post run using the ∆∆Ct method^58^. Fold change values were log_2_-transformed prior to determining the statistical significance between groups via ANOVA with Dunnett’s post-test in Prism (GraphPad).

### Phenoloxidase (PO) enzymatic activity assay

Determination of PO enzymatic activity was adapted from Sadd *et al*.^59^. Briefly, mosquitoes were collected at 24 h and 6d post-infection with two whole mosquitoes pooled per sample (20 samples per treatment at 24 h PI and 10 samples per treatment at 6d PI). Phenoloxidase activity assays were performed by first homogenizing samples with the TissueLyser II (Qiagen) for 30 s with a 2.4 mm bead and 50 µl of 1x PBS. Homogenates were quickly centrifuged at 3000 rpm, 4 °C, for 5 min, and 35 µl of the supernatant transferred to a new vial, snap frozen in liquid nitrogen and stored at −80 °C for subsequent analyses. Phenoloxidase activity was evaluated in a 96-well plate by mixing 15 µl of mosquito homogenate with 20 µl of PBS and 140 µl of molecular-grade water. Reaction was started by adding 20 µl of L-Dopa (4 mg per mL H_2_O; 3,4 dihyroxy-L-phenylalanine) and plates shaken for 5 s at 30 °C in a spectrophotometer (Multiskan GO®, Thermal Scientific). Changes in absorbance were measured at 490 nm every 15 s with 5 s shaking between reads. PO enzymatic activity was determined from the slope (V_max_) of the reaction curve and from over 160 readings. Each sample was read in duplicate and the average used in further analyses. In total, three independent experiments were conducted, each time employing a fresh batch of fungi and new cohorts of mosquitoes.

### Statistical analyses

Statistical analyses of experimental assays were conducted using GraphPad Prism 7 (GraphPad). Survival after fungal infections were analyzed using Kaplan-Meier with Log-rank tests. Statistical significance was assessed at *P* < 0.05 and its strength is represented with asterisks (*P < 0.05; **P < 0.01; ***P < 0.001). Unless indicated otherwise, the error bars represent the standard error of the mean and the type of test used is indicated in the respective figure legend. Probit analysis was used to determine the LC_50_, LT_50_ and LT_95_ values using SAS 9.4 statistical package.

### Data availability

All data generated or analyzed during this study are included in this published article (and its Supplementary Information files).

## Electronic supplementary material

Supplementary material
