# Supplementary material for: Strain-specific pathogenicity and subversion of phenoloxidase activity in the mosquito Aedes aegypti by members of the fungal entomopathogenic genus Isaria
Source: Sci Rep. 2018 Jul 2;8:9896. doi: 10.1038/s41598-018-28210-6 (PMC6028645; doi:10.1038/s41598-018-28210-6)
Supplement: Supplementary file 1 — Supplementary material [file 41598_2018_28210_MOESM1_ESM.pdf]

Strain-specific pathogenicity and subversion of phenoloxidase activity in the mosquito *Aedes aegypti* by members of the fungal entomopathogenic genus *Isaria.*

José L. Ramirez,[1]*, Ephantus J. Muturi[1], Christopher Dunlap[1], and Alejandro P. Rooney[1]

Crop Bioprotection Research Unit, National Center for Agricultural Utilization Research, Agricultural Research Service, United States Department of Agriculture, Peoria, Illinois, USA[1]

SUPPLEMENTARY INFORMATION

Table S1 .- Percent sporulation from mosquito cadavers collected from each of the infectious doses applied to the mosquitoes.

	**% Sporulation**						
**Fungal Dose**	***I. tenuipes*** (ARSEF 3939)	***I. amoenerosea*** (CBS 107.73)	***I. javanica*** (ARSEF 5874)	***I. cateniannulata*** (ARSEF 6241)	***I. amoenerosea*** (ARSEF 741)	***I. flavovirescens*** (ARSEF 1686)	***I. poprawskii*** (ARSEF 7028)
5.1×10^1	0	0	0	60	13	40	25
5.1×10^2	50	33	33	71	100	33	50
5.1×10^3	0	75	100	100	86	50	-
5.1×10^4	75	100	87	79	81	67	100
5.1×10^5	89	92	91	93	-	100	100

Table S2. Primer sequences used in qPCR.

Gene ID	Gene Name	Forward Primer Sequence	Reverse Primer Sequence	Reference
AAEL011763	PPO3	TTGCTAAGAAAGATCGCCGT	AATCGGTGCCTGTAGATTGG	1
AAEL013492	PPO5	ATGACCGACTGGAAAACTGG	ACGTTTGGCCTCCATACTTG	1
AAEL000087	TEP22	TACCTCCTGGTACGTGACCG	TACTTCACCTCGCTTTATCG	1
AAEL003396	Rps49	GCTATGACAAGCTTGCCCCCA	TCATCAGCACCTCCAGCT	2
Fungal load	18s rRNA	AGATACCGTCGTAGTCTTAACCATAAACT	TTCAGCCTTGCGACCATACT	3
AAEL004175	Rps17	TCCGTGGTATCTCCATCAAGCT	CACTTCCGGCACGTAGTTGTC	4
AAEL007696	Rel1	TGGTGGTGGTGTCCTGCGTAAC	CTGCCTGGCGTGACCGTATCC	5
AAEL000598	CecD	ATGAACTTCACTAAGCTGTT	TCATTTTCCAATCGCTTTTAT	5
AAEL009692	STAT	CACACAAAAAGGACGAAGCA	TCCAGTTCCCCTAAAGCTCA	2
AAEL003841	DefA	TAGTGCTTGTGCTGCTCATTG	TAGGTATAAGTTGCTCGAAGG	6

Supplementary information references

1 Wang, Y. H. *et al.* A Critical Role for CLSP2 in the Modulation of Antifungal Immune Response in Mosquitoes. *PLoS Pathog* **11**, e1004931, doi:10.1371/journal.ppat.1004931 (2015).

2 Barletta, A. B. F., Silva, M. C. L. N. & Sorgine, M. H. F. Validation of Aedes aegypti Aag-2 cells as a model for insect immune studies. *Parasites & Vectors* **5**, 148, doi:10.1186/1756-3305-5-148 (2012).

3 Bell, A. S., Blanford, S., Jenkins, N., Thomas, M. B. & Read, A. F. Real-time quantitative PCR for analysis of candidate fungal biopesticides against malaria: technique validation and first applications. *J Invertebr Pathol* **100**, 160-168, doi:10.1016/j.jip.2009.01.006 (2009).

4 Joubert, D. A. *et al.* Establishment of a <italic>Wolbachia</italic> Superinfection in <italic>Aedes aegypti</italic> Mosquitoes as a Potential Approach for Future Resistance Management. *PLoS Pathog* **12**, e1005434, doi:10.1371/journal.ppat.1005434 (2016).

5 Pan, X. *et al.* Wolbachia induces reactive oxygen species (ROS)-dependent activation of the Toll pathway to control dengue virus in the mosquito Aedes aegypti. *Proc Natl Acad Sci U S A* **109**, doi:10.1073/pnas.1116932108 (2012).

6 Wang, Y.-H. *et al.* A Critical Role for CLSP2 in the Modulation of Antifungal Immune Response in Mosquitoes. *PLOS Pathogens* **11**, e1004931, doi:10.1371/journal.ppat.1004931 (2015).
